# Identification of novel suggestive loci for high-grade myopia in Polish families

**Published:** 2011-07-22

**Authors:** Malgorzata Rydzanicz, Swapan K. Nath, Celi Sun, Monika Podfigurna-Musielak, Agata Frajdenberg, Malgorzata Mrugacz, Daniel Winters, Uppala Ratnamala, Uppala Radhakrishna, Bassem A. Bejjani, Marzena Gajecka

**Affiliations:** 1 Institute of Human Genetics, Polish Academy of Sciences, Poznan, Poland; 2 Arthritis and Immunology Research Program, Oklahoma Medical Research Foundation, Oklahoma City, OK; 3 Department of Ophthalmology, Leszno Hospital, Leszno, Poland; 4 Department of Ophthalmology, Marcinkowski University of Medical Sciences, Poznan, Poland; 5 Namsos Hospital, Department of Ophthalmology, Namsos, Norway; 6 University Hospital in Linköping, Department of Ophthalmology, Linköping, Sweden; 7 Department of Pediatric Ophthalmology, Medical University of Bialystok, Bialystok, Poland; 8 School of Molecular Biosciences, Washington State University, Spokane, WA; 9 Department of Surgery-Transplant, University of Nebraska Medical Center, Omaha, NE; 10 Signature Genomic Laboratories, LLC, Spokane, WA; 11 Basic Medical Sciences Program, WWAMI, Spokane, WA

## Abstract

**Purpose:**

Myopia is the most common human eye disorder with complex genetic and environmental causes. To date, several myopia loci have been identified in families of different geographic origin. However, no causative gene(s) have yet been identified. The aim of this study was the characterization of Polish families with high-grade myopia, including genetic analysis.

**Methods:**

Forty-two multiplex Polish families with non–syndromic high-grade myopia participated in the study. All family members underwent detailed ophthalmic examination and high-grade myopia was defined as ≤-6.0 diopters (D) based on the spherical refractive error. A genome-wide single nucleotide polymorphism (SNP)-based high-density linkage scan was performed using *Affym*etrix Human SNP Array 6.0 on a selected family (HM-32) with multiple affected individuals.

**Results:**

Nonparametric linkage analysis identified three novel loci in family HM-32 at chromosome 7p22.1–7p21.1 ([NPL] 8.26; p=0.006), chromosome 7p12.3–7p11.2 ([NPL] 8.23; p=0.006), and chromosome 12p12.3–12p12.1 ([NPL] 8.02; p=0.006), respectively. The effect of linkage disequilibrium on linkage due to dense SNP map was addressed by systematically pruning SNPs from the linkage panel.

**Conclusions:**

Haplotype analysis with informative crossovers in affected individuals defined a 12.2; 10.9; and 9.5 Mb genomic regions for high-grade myopia spanned between SNP markers rs11977885/rs10950639, rs11770622/rs9719399, and rs4763417/rs10842388 on chromosomes 7p22.1–7p21.1, 7p12.3–7p11.2, and 12p12.3–12p12.1, respectively.

## Introduction

Myopia, also known as shortsightedness, is the most common eye disorder worldwide. In myopic subjects, the image of distant objects falls in front of the retina, either as the eye is too long (axial myopia), the cornea is too convex or the index of refraction of the lens is too high (refractive myopia) [1]. The myopic eye is generally vulnerable and persons with ≤-6.0 diopters (D) are more liable to a wide range of ocular pathologies. The development of high-grade myopia involves anterior-posterior enlargement of the eye, scleral thinning, changes in the diameter of scleral collagen fibrils, and frequent detachment of the retina resulting from stress related with axial elongation [2].

The estimated prevalence of high grade myopia is ~2.5 to 9.6% in the elderly world population [3,4]. However, its highest prevalence rates are in Asians, in whom almost 50 to 80% of the adult populations are myopic [5-7]. Recent population-based studies suggest that the prevalence is increasing, specifically in Asian populations [8,9]. The frequency of myopia in the Polish population is unknown, and there is a paucity of data about the epidemiology of high-myopia in Poland. Until the present study, no analysis has yet been made on familial high-grade myopia. However, in Poland the main cause of blindness and ~12% childhood visual impairment is due to high-grade myopia [10].

Myopia may be of diverse etiology, including environmental and genetic factors [11-17]. However, high-grade myopia is highly heritable and genetic predispositions seem to be a dominant factor of its development and progression [18,19]. Families with autosomal dominant, autosomal recessive, and X-linked inheritance of high-grade myopia have been described, though the majority of the reports deal with the autosomal dominant form [20-28]. Although there were no obvious phenotypic differences between the affected subjects of families used in the previously published linkage analyses, the data were inconsistent, suggesting genetic heterogeneity among populations of different geographic origin [20,23]. Several studies have demonstrated a significant genetic component in the familial aggregation of high-grade and/or moderate myopia. Up to 17 loci on different chromosomal regions have been identified [20,22,24-37]. Additionally, increased incidence of single nucleotide polymorphism (SNP)s association between the insulin-like growth factor 1 (*IGF-1*) gene and high-grade myopia [38], and polymorphisms in the promoter regions of matrix metalloproteinase (MMP) genes were reported [39]. Moreover, two recent independent genome-wide association studies conducted on large cohorts of refractive error patients identified loci at chromosome 15q14 and 15q25 and suggest that the genetic variance in refractive errors is mostly determined by multiple variants with a low to moderate penetrance [40,41].

In this study we present clinical characteristics of forty two Polish families with non-syndromic high-grade myopia and the results of a high-density SNP-based linkage analysis for one selected large high-grade myopia family with multiple affected and normal individuals. Our findings provided evidence of suggestive linkage at three distinct novel loci on chromosome 7p22.1–7p21.3, 7p12.3–7p11.2, and 12p12.3–12p12.1 in the analyzed family.

## Methods

### Recruitment and clinical evaluation of high-grade myopia families

The study population consisted of 42 multiplex high-grade myopia families from Poland, who were ascertained at three independent Polish institutions: 1) Department of Ophthalmology, Marcinkowski University of Medical Sciences, in Poznan, 2) Department of Pediatric Ophthalmology, University of Medical Sciences in Bialystok, and 3) Department of Ophthalmology, Hospital, Leszno. A constant clinical evaluation procedure was applied at all clinical sites. Informed consent was obtained from all study subjects after the possible consequences of participating in the study were explained, in accordance with the Declaration of Helsinki.

All study subjects underwent a detailed ophthalmic evaluation using computer-assisted equipment included: a visual acuity testing, best-corrected visual acuity testing, a slit lamp evaluation, intraocular pressure examination, fundoscopy, axial length determination, keratometry and refractometry. Biometric axial length (including anterior-chamber depth, lens thickness, and total axial length) was measured using ultrasonography (A, OPTOPOL, Desmin F/H, version 2.06.21). In children ≤15 years old, the refractive error was measured with an autorefractor after cycloplegia. A complete questionnaire was filled for each subject with clinical and family history.

To minimize misclassification, clear diagnostic criteria were established for all high-grade myopia study subjects including spherical refractive error analysis. The subjects were classified into three groups, 1) Affected individuals with high-grade myopia, 2) Individuals with an unknown status and 3) Unaffected persons. All affected individuals showed: 1) bilateral axial high-grade myopia, in excess of or equal to −6.0 D (≤-6.0 D) for at least one eye and in excess of or equal to −5.0 D (≤-5.0 D) for the second eye; 2) a history of onset of myopia at age ≤15 years, and 3) individual with affected status while high-grade myopia was identified in multiple members of their family in different generations. Individuals who were classified as unknown were: 1) all children ≤15 years unless they fulfill criteria for affected status as specified above, or 2) individuals who have myopia with −6.0 D < X ≤ −4.0 D, or 3) individuals, with a refractive error of ≤-6.0 D for one eye and a refractive error >-5.0 D for the second eye, or 4) individuals with late age of onset (>15 years). All the remaining were treated as unaffected as neither of them were classified as affected nor unknown for the analysis.

For all 42 Polish HM families we have performed the analysis using microsatellite markers to exclude or confirm linkage with known high myopia loci (data not shown). In all families previously suggested candidate loci/genes for high myopia were excluded (data not shown).

### Statistical analysis in clinical evaluation

Differences in ophthalmic parameters obtained for respective groups, as well as comparison of age were analyzed by the Kruskal–Wallis test [42]. Gender distribution was calculated by χ^2^ test. All analyzed features were compared among groups according to the scheme: affected versus unaffected, affected versus unknown and unaffected versus unknown. The differences between examined groups were considered significant if the value of probability (p) did not exceed 0.05. Axial length in affected individuals helped reveal whether a patient had corrective surgery in the past. Affected subjects who underwent corrective surgery were not included in analysis of mean refraction value for high-grade myopes versus non-highly myopic subjects.

### Genome-wide genotyping in family HM-32

The family HM-32 was chosen for genome-wide genotyping analysis. The selected pedigree was the largest, multigenerational, representative family with many available family members, including patients with high-grade myopia, as well as unaffected relatives.

A genome-wide SNP-based high-density linkage scan was performed using the Affymetrix Human SNP array 6.0 (Affymetrix Inc., Santa Clara, CA) which features 1.8 million genetic markers, including 906,600 SNPs and 946,000 probes for the detection of copy number variation. The assay was performed with 500 ng of genomic DNA, and more than 99% of the SNPs were determined unequivocally for each sample. Scanned images were processed with gene microarray software (Affymetrix) and the data was analyzed (GDAS ver. 2, software; GeneChip Data Analysis; Affymetrix). PEDCHECK [43] was used to identify Mendelian inconsistencies, and the MERLIN [44] program was used to detect double recombination events over short genetic distances that were probably due to genotyping errors. After the quality control (QC) of the raw genotype data which deleted SNPs with missing genotypes and all SNPs at which all individuals have the ‘BB’ or ‘AA’ genotypes, there were a total 550,441 SNPs left for analysis. The genotypes and the markers generated from the Affymetrix 6.0 platform were so dense that linkage disequilibrium between many markers will result in severe biases in linkage calculations. Since we analyzed one family with 16 samples genotypes available, we used the allele frequencies from HapMap CEU population for the analysis. To prevent bias from linkage disequilibrium (LD) in linkage calculations, we first created genotype subset with 4,417 SNPs for the genome wide linkage scan by selecting one SNP from every 100,000 bases in the QC genotype data set. While selecting SNPs, the minor allele frequency (MAF) >1% at each SNP was also a criterion for selection. To maximize the heterozygosity, we always selected the SNPs with high MAF. In a case of identified candidate interval(s) further analysis was performed, selecting one SNP from every two SNPs from candidate interval(s) for the analysis to eliminate the bias from LD. Moreover, linkage analysis was also done by another way to account for LD effect by using genetic distances, selecting one SNP per 0.5 cM, 1 cM, 1.5 cM, and 2 cM for the linkage analysis, respectively.

SNP genotype data were imported into the linkage-analysis programs GENEHUNTER [45] and MERLIN [44]. In the initial genome scan, evidence of linkage was assessed with a nonparametric, penetrance-independent, affected-only, and allele-sharing analysis (Z-mean from MERLIN and nonparametric linkage (NPL) from GENEHUNTER). With MERLIN, one can convert this into a nonparametric logarithm (base 10) of odds (LOD*) score by maximizing the likelihood with respect to a scalar parameter, δ, that measures the amount of excess sharing of identical-by-descent alleles among affected relatives (with δ=0) corresponding to the null hypothesis of no linkage [46]. We used the *S*_all_ scoring function and the exponential allele-sharing model to generate the relevant linkage statistic. When significant evidence of linkage was found by exceeding the predetermined threshold (p<0.01), two-point as well as multipoint LOD scores maximized over various plausible genetic model parameters were calculated. For the parametric linkage analysis the best model was estimated as an autosomal dominant mode of inheritance with reduced penetrance (0.6) and phenocopy rate (0.01) and a disease allele frequency of 0.0001. In addition, for the parametric linkage analysis an affected only analysis was performed under an autosomal dominant mode of inheritance allowing for phenocopies. Genetic map distances were derived from the Rutgers combined linkage-physical map of the human genome [47], either directly or by interpolation. Haplotypes were reconstructed using both GENEHUNTER and SIMWALK2 programs [48,49].

## Results

### Clinical and demographic characteristics of studied families

**Figure 1:**
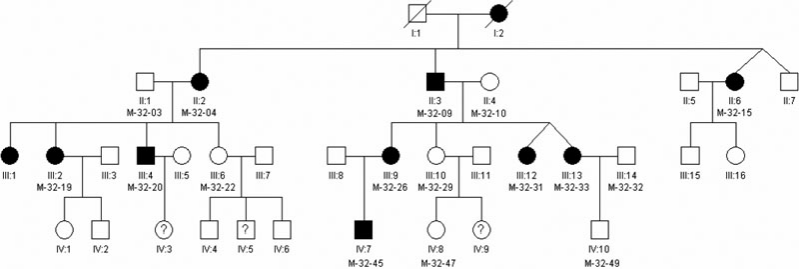
Pedigree of family HM-32 with high myopia. Blackened symbols: individuals with high myopia; unblackened symbols: unaffected individuals; symbols with question mark: individuals with unknown disease status. Individuals used in the linkage analysis are numbered under their symbols in the pedigree.

**Figure 2:**
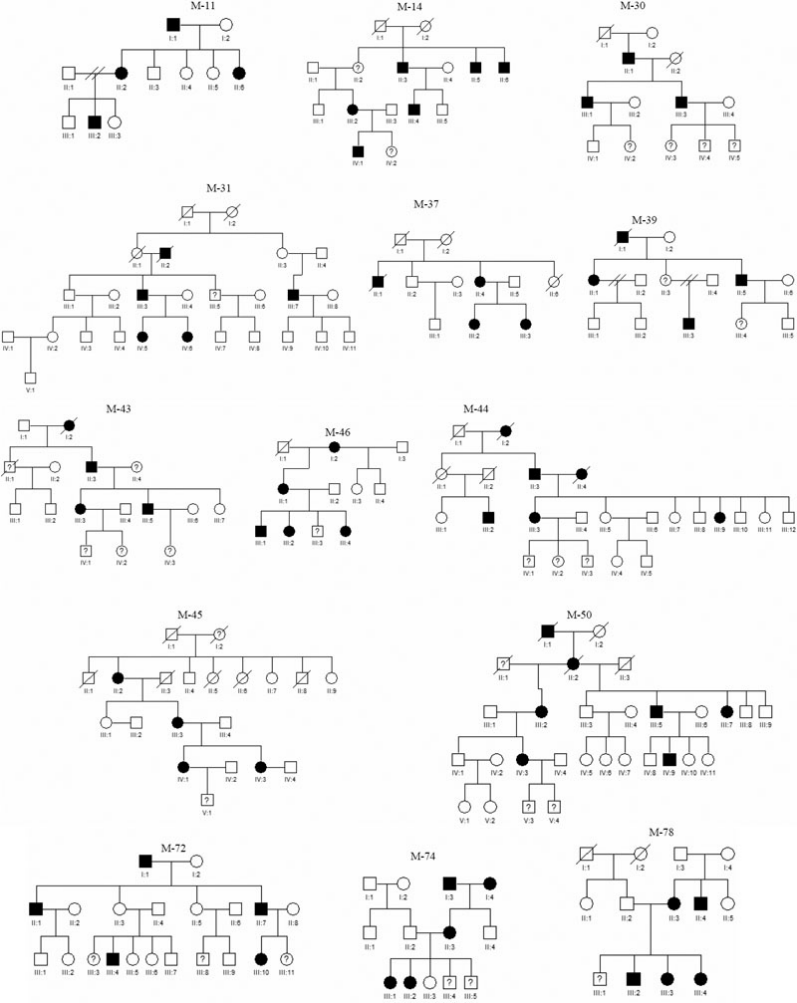
Pedigrees of 14 of 42 Polish families, with familial high myopia. Filled symbols: individuals with high myopia; open symbols: unaffected individuals; symbols with question mark: individuals with unknown disease status.

The forty-two large Polish pedigrees enrolled in the study, had families with five-generation (n=10), four-generations (n=7), three-generations (n=27), and two-generations (n=7) with an average number of individuals in each generation per family of 3.5 (Figure 1, Figure 2, and Appendix 1). The mean family size was 8.3 individuals (range 3–45), with the average affected individuals per family of 3.0 (range 2–10), unaffected 4.1 (range 1–28) and unknown status 0.8 (range 1–7). The specified information was based on individuals who underwent an ophthalmic examination.

A complete eye examination was performed for 331 participated individuals. In accordance with our classification criteria, 128 individuals were considered as affected, 171 as unaffected, and 32 with unknown status. The characteristics and details of the ophthalmic examinations in particular study groups are given in Table 1. Additionally, Appendix 2 shows detailed clinical findings in family HM-32. Spherical refractive error alone (without cylindrical refractive error) was enough to classify 128 affected individuals in this category.

**Table 1:** Clinical characteristics of examined individuals in 42 high-grade myopia Polish families.

**Clinical characteristics**	**Affected**			**Unaffected**			**Unknown**		
No. of individuals	128			171			32		
**Age at Examination [y]**									
Range	5–87			3–86			3–81		
Mean age (±SD)	40.2 (±20.43)			37.7 (±18.75)			27.0 (±22.26)		
**Age of Onset [y]**									
Range	2–15			—			—		
Mean age (±SD)	8.21 (±3.40)			—			—		
**Gender**									
F	77 (60.2%)			95 (55.6%)			13 (40.6%)		
M	51 (39.8%)			76 (44.4%)			19 (59.4%)		
**Spherical refractive error [D]**									
	OD	OS	OD+OS	OD	OS	OD+OS	OD	OS	OD+OS
n	118	120	238	170	169	339	32	32	64
Minimum	−20.75	−21.00	−21.00	−3.50	−3.75	−3.75	−5.25	−6.00	−6.00
25% Percentile	−11.00	−11.00	−11.00	−0.50	−0.50	−0.50	−4.50	−4.50	−4.50
Median	−8.00	−7.75	−7.87	0.00	0.00	0.00	−4.00	−3.87	−4.00
75% Percentile	−6.75	−6.50	−6.50	0.50	0.50	0.50	−0.62	−0.50	−0.50
Maximum	−5.00	−5.00	−5.00	4.50	6.00	6.00	0.50	0.75	0.75
Mean	−9.34	−9.29	−9.32	−0.03	0.02	0.00	−2.79	−2.86	−2.82
(±SD)	(±3.95)	(±3.84)	(±3.89)	(±1.22)	(±1.25)	(±1.23)	(±1.94)	(±2.13)	(±2.02)
**Cylindrical refractive error [D]**									
	OD	OS	OD+OS	OD	OS	OD+OS	OD	OS	OD+OS
n	117	119	236	170	169	339	32	32	64
Minimum	−6.25	−5.00	−6.25	−2.75	−4.00	−4.00	−1.00	−1.25	−1.25
25% Percentile	−1.50	−1.50	−1.50	−0.62	−0.62	−0.50	−0.50	−0.50	−0.50
Median	−0.50	−0.50	−0.50	−0.25	−0.25	−0.25	0.00	0.00	0.00
75% Percentile	0.00	0.00	0.00	0.00	0.00	0.00	0.00	0.00	0.00
Maximum	1.50	1.500	1.500	2.00	1.75	2.00	1.75	2.25	2.25
Mean	−0.90	−0.84	−0.87	−0.37	−0.35	−0.36	−0.23	0.12	−0.17
(±SD)	(±1.34)	(±1.15)	(±1.24)	(±0.62)	(±0.74)	(±0.68)	(±0.52)	(±0.62)	(±0.57)
**Spherical equivalent [D]**									
	OD	OS	OD+OS	OD	OS	OD+OS	OD	OS	OD+OS
n	117	118	235	170	169	339	32	32	64
Minimum	−21.0	−21.00	−21.00	−4.50	−3.75	−4.50	−5.25	−6.00	−6.00
25% Percentile	−11.82	−11.50	−11.62	−0.75	−0.50	−0.75	−4.50	−4.56	−4.50
Median	−8.38	−8.00	−8.25	−0.25	0.00	−0.12	−3.75	−4.00	−3.87
75% Percentile	−6.50	−6.75	−6.50	0.31	0.50	0.37	−0.62	−0.50	−0.50
Maximum	−4.75	−5.00	−4.75	4.00	5.62	5.62	0.00	1.50	1.50
Mean	−9.72	−9.63	−9.69	−0.24	−0.16	−0.19	−2.90	−2.93	−2.91
(±SD)	(±4.09)	(±3.91)	(±4.00)	(±1.21)	(±1.24)	(±1.22)	(±1.92)	(±2.11)	(±2.00)
**Axial length [mm]**									
	OD	OS	OD+OS	OD	OS	OD+OS	OD	OS	OD+OS
n	127	127	254	166	166	332	31	31	62
Minimum	23.28	23.42	23.28	21.15	20.91	20.91	22.31	21.53	21.53
25% Percentile	26.00	26.01	26.01	22.83	22.78	22.82	23.52	23.42	23.45
Median	26.95	26.95	26.95	23.32	23.40	23.37	24.17	24.40	24.18
75% Percentile	28.12	28.28	28.21	23.90	23.95	23.92	24.92	24.89	24.91
Maximum	36.41	35.51	36.41	25.69	25.80	25.80	27.18	26.48	27.18
Mean	27.26	27.27	27.27	23.35	23.37	23.36	24.34	24.28	24.31
(±SD)	(±2.09)	(±1.96)	(±2.03)	(±0.81)	(±0.85)	(±0.83)	(±1.14)	(±1.16)	(±1.14)
**Keratometry [D]**									
	OD	OS	OD+OS	OD	OS	OD+OS	OD	OS	OD+OS
n	109	110	219	140	140	280	24	24	48
Minimum	40.00	40.00	40.00	38.50	38.50	38.50	40.25	41.18	40.25
25% Percentile	42.50	42.92	42.82	42.50	42.80	42.75	43.08	43.10	43.08
Median	43.87	43.93	43.87	43.75	43.75	43.75	43.87	43.75	43.84
75% Percentile	44.62	44.87	44.75	44.37	44.50	44.50	44.25	44.59	44.31
Maximum	47.75	47.25	47.75	47.00	47.00	47	45.62	45.75	45.75
Mean	43.74	43.82	43.78	43.50	43.57	43.53	43.74	43.76	43.75
(±SD)	(±1.61)	(±1.51)	(±1.56)	(±1.43)	(±1.45)	(±1.44)	(±1.08)	(±1.04)	(±1.05)

There are several individuals in this study with unknown disease status, which partly due to the involvement of children <15 years. In 50% of cases the unknown disease status is due to the inclusion of individuals with an average spherical refractive error (SPH) ranges between −6.0 D < X ≤ −4.0 D. For example, in families 10, 14, 39 and 75, individuals 10-III: 3, 14-II: 2, 39-III: 4 and 75-I: 4 presented SPH as follow: −4.75/-4.75 D, −4.5/-5.25 D, −4.25/-4.25 D and −4.5/-6.0 D, respectively.

Based on medical records and/or self-reports the average age of onset in myopic subjects was ~8 years (range 2–15). Affected females had slightly earlier onset than affected males (7.78 years, range 2–14 versus 8.88 years, range 2–15, respectively), however the difference was not statistically significant (p=0.077). Some of the affecteds were found with various associated anomalies including glaucoma (11.7%; n=15), cataract (4.7%; n=6), retinal detachment (RD; 5.5%; n=7), and RD in both eyes (n=2). In both unknown and unaffected individuals no other anomalies were identified except one normal individual with glaucoma (0.6%).

We have found statistically significant differences in spherical refractive error, spherical equivalent refractive error (SE) and axial length (AL) between the studied groups (p<0.001; Appendix 3). In affected subjects the average SPH was −9.32 D (±3.89), where the median value was −7.87 D, compared with −2.82 D (±2.02, median −4.00 D) for unknown and 0.00 D (±1.23, median 0.00 D) for unaffected individuals. The average spherical equivalent refractive error of the affected was −9.69 D (±4.00, median −8.25 D), where for unknown and unaffected respectively, −2.91 D (±2.00, median −3.87 D) and −0.19 D (±1.22, median −0.12 D). The mean AL (27.27±2.03, median 26.95 mm) of high-grade myopic eyes was significantly higher than observed for unknown (24.31±1.14, median 24.18 mm) and unaffected individuals (23.36±0.83, median 23.37 mm; Table 1 and Appendix 3). There was no difference in keratometry among the studied groups.

### Genome-wide genotyping in family HM-32

**Figure 3:**
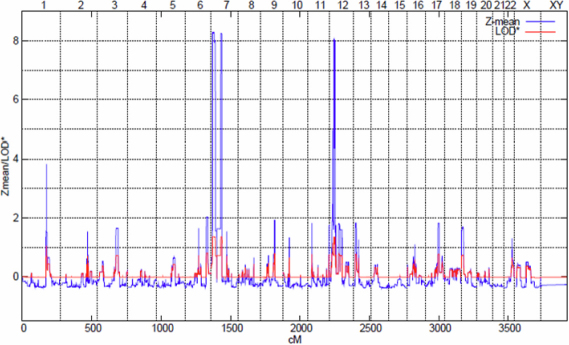
Results of the multipoint nonparametric linkage analysis for family HM-32. The x-axis represents the chromosomal location for each of the 22 autosomes, and the y-axis represents the Z mean/LOD. The highest peak are at chromosome 7p22.1–7p21.1 (nonparametric linkage [NPL] 8.26; p=0.006), 7p12.3–7p11.2 ([NPL] 8.23; p=0.006), and 12p12.3–12p12.1 ([NPL] 8.02; p=0.006).

**Figure 4:**
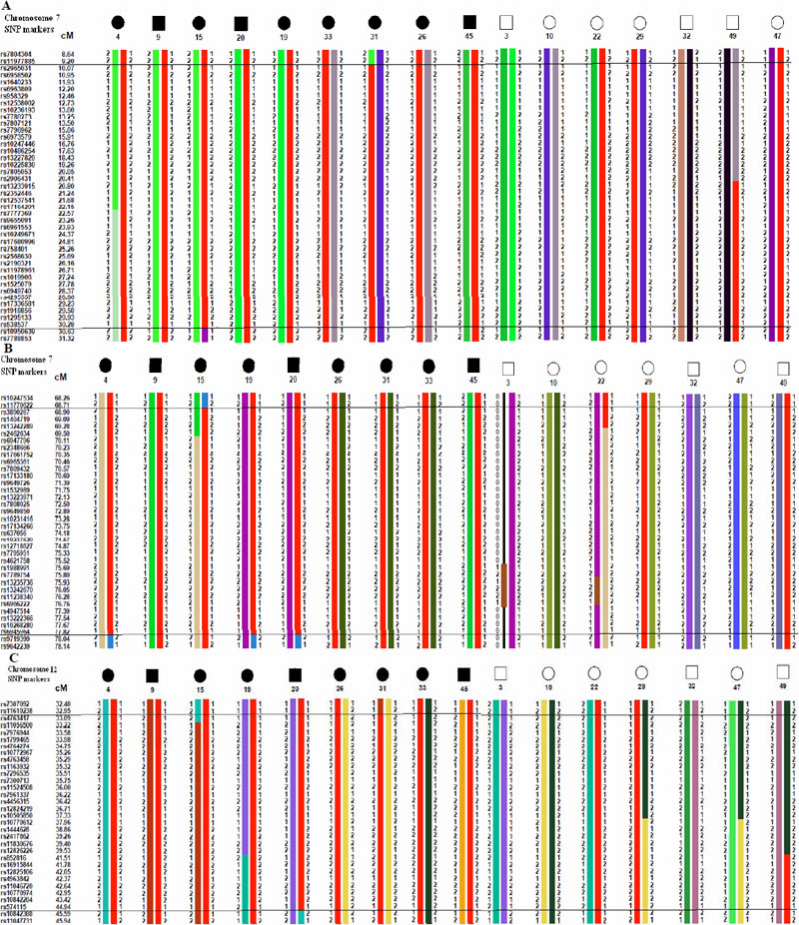
Genotypes and haplotypes of chromosomes 7p22.1–7p21.1, 7p12.3–7p11.2, and 12p12.3–12p12.1. Haplotypes associated with affected status are shown in red. Haplotype analysis showed that the cosegregating segment of the MYP loci in family HM-32 was between markers rs11977885/rs10950639, rs11770622/rs9719399 and rs4763417/rs10842388 on chromosomes **A**: 7p22.1–7p21.1, **B**: 7p12.3–7p11.2, and **C**: 12p12.3–12p12.1, respectively.

Genome-wide (SNP)-based high density linkage analysis in a large high-grade myopia family (HM-32) has shown evidence for susceptibility loci on chromosomes 7p22.1–7p21.1 ([NPL] 8.26; p=0.006); 7p12.3–7p11.2 ([NPL] 8.23; p=0.006) and 12p12.3–12p12.1 ([NPL] 8.02; p=0.006: Table 2 and Figure 3) suspected to be implicated in high-grade myopia in the present family. These results were also supported by multipoint parametric linkage analysis with maximum multipoint LOD scores of 1.33, 2.12, and 1.81 for the respective chromosomal regions. Haplotypes were constructed using over 30 informative SNP markers on three variant linked chromosomal regions. Segregation analysis identified a risk haplotypes that was shared by all affected individuals at three loci and was not found in any of the unrelated unaffected spouses. Critical recombination events that occurred in two affected patients M-32–31 and M-32–15 allowed us to define a 21.4 cM disease interval delimited by SNPs rs11977885 and rs10950639 on chromosome 7p22.1–7p21.1 (Figure 4A). Informative recombination events in affected individuals M-32–04, M-32–15, M-32–19, and M-32–20, confined the myopia candidate locus on chromosome 7p12.3–7p11.2 to a region of 9.3 cM between SNPs rs11770622 and rs9719399 (Figure 4B). Similar haplotype analysis and critical recombination events across the affected family members M-32–15 and M-32–20 on chromosome 12p12.3–12p12.1 narrowed the genomic region to 12.6 cM (Figure 4C). The area is bordered by proximal marker rs4763417 and distal marker rs10842388 (Appendix 4A-C). We also tried another way to prune out the LD by using genetic distances, selecting one SNP per 0.5 cM, 1 cM, 1.5 cM and 2 cM for the linkage analysis. The results were similar compared to what we described earlier except for the second interval 68 cM- 80 cM on chromosome 7p13–7p11.2, which shows a change from 0.5 cM to 1 cM, but still remains significant P value (Appendix 5).

**Table 2:** Linkage analysis results: Three susceptibility loci Identified in HM-32.

					**Non-parametric**			
**Cytogenetic**	**Decode map (cM)**	**SNP marker**	**Physpos (bp)**	**Parametric LOD**	**npl_Zmean**	**NPL**	**p value**	**npl LOD**
7p22.1	9.20	rs11977885	4653045	0.904	4.26	4.26	0.013	1.09
7p22.1	11.93	rs1640233	5609671	1.316	8.19	8.19	0.006	1.36
7p22.1	12.73	rs12538002	6577858	1.317	8.26	8.26	0.006	1.36
7p21.3	13.50	rs7807121	7520065	1.319	8.25	8.25	0.006	1.36
7p21.3	16.76	rs10247446	8444937	1.326	8.21	8.21	0.006	1.36
7p21.3	18.43	rs13227829	9071553	1.201	8.24	8.24	0.006	1.36
7p21.3	20.05	rs7805053	9690241	1.035	8.25	8.25	0.006	1.36
7p21.3	22.15	rs17164201	11282715	0.928	8.05	8.05	0.006	1.35
7p21.3	23.26	rs9655091	11892872	0.925	7.93	7.93	0.006	1.34
7p21.3	24.37	rs10249671	12514442	0.920	7.86	7.86	0.006	1.34
7p21.3	25.26	rs758401	13135380	0.914	7.78	7.78	0.007	1.34
7p21.2	26.16	rs2190321	13750336	0.907	7.67	7.67	0.007	1.33
7p21.2	27.24	rs1019906	14369309	0.892	7.46	7.46	0.007	1.32
7p21.1	29.23	rs17336581	15658618	0.803	6.37	6.37	0.008	1.25
7p21.1	30.28	rs538537	16584971	0.609	4.54	4.54	0.012	1.12
7p21.1	30.63	rs10950639	16892043	−2.812	1.51	1.51	0.040	0.70
7p13	68.71	rs11770622	46163791	−2.225	1.57	1.57	0.040	0.71
7p12.3	69.09	rs1404719	46782062	1.536	5.87	5.87	0.009	1.22
7p12.3	69.50	rs2462634	47400153	2.082	7.63	7.63	0.007	1.33
7p12.3	70.23	rs2348666	48045318	2.115	8.11	8.11	0.006	1.35
7p12.3	70.46	rs6965361	48664499	2.117	8.14	8.14	0.006	1.35
7p12.2	71.75	rs1532989	49912251	2.121	8.21	8.21	0.006	1.36
7p12.2	72.50	rs7808025	50544397	2.123	8.23	8.23	0.006	1.36
7p12.1	73.28	rs10231416	51181101	2.123	8.22	8.22	0.006	1.36
7p12.1	74.18	rs637056	51797737	2.122	8.22	8.22	0.006	1.36
7p12.1	74.87	rs12718627	52417115	2.122	8.21	8.21	0.006	1.36
7p11.2	75.80	rs7789754	54015519	2.114	8.06	8.06	0.006	1.35
7p11.2	76.05	rs13242670	54673192	2.110	7.98	7.98	0.006	1.35
7p11.2	76.76	rs6966222	55301496	2.031	6.65	6.65	0.008	1.27
7p11.2	77.54	rs13222366	55967196	1.838	4.23	4.23	0.013	1.09
7p11.2	77.82	rs6945964	56654367	1.467	1.76	1.76	0.030	0.75
7p11.2	78.04	rs9719399	57159680	−1.882	−0.09	−0.09	0.500	0.00
12p12.3	32.95	rs11610238	14866988	1.773	7.44	7.44	0.007	1.32
12p12.3	33.22	rs11056500	15500344	1.808	7.92	7.92	0.006	1.34
12p12.3	33.98	rs1799465	16176985	1.811	7.96	7.96	0.006	1.35
12p12.3	35.26	rs10772967	16852267	1.814	8.02	8.02	0.006	1.35
12p12.3	35.32	rs1163932	17492875	1.814	8.02	8.02	0.006	1.35
12p12.3	35.75	rs7300713	18175590	1.814	8.02	8.02	0.006	1.35
12p12.3	36.22	rs7961337	18855833	1.814	8.02	8.02	0.006	1.35
12p12.3	36.71	rs12824219	19488622	1.814	8.02	8.02	0.006	1.35
12p12.2	37.96	rs10770612	20121906	1.791	8.01	8.01	0.006	1.35
12p12.2	39.26	rs2417862	20761532	1.727	7.79	7.79	0.007	1.34
12p12.1	39.53	rs12826226	21414787	1.706	7.64	7.64	0.007	1.33
12p12.1	41.78	rs16915844	22053236	1.494	6.23	6.23	0.008	1.24
12p12.1	42.37	rs4963842	22727319	1.378	5.39	5.39	0.010	1.19
12p12.1	42.95	rs10770974	23348839	1.251	4.63	4.63	0.011	1.12
12p12.1	44.94	rs574115	24383760	0.442	2.01	2.01	0.030	0.80
12p12.1	45.59	rs10842388	24693591	−2.603	1.22	1.22	0.040	0.63

The SNP-based copy number analyses did not reveal any variation in the linked genomic regions.

## Discussion

Myopia is the most common ocular disorder in all populations and the incidence is increasing in all parts of the world [3]. After the first genetic linkage studies of X-linked syndromic form of myopia, many loci were mapped in highly-selected families that aggregated severe forms of myopia in diverse populations of various geographic origins and some attempted to replicate the data [20,22,24,50-53]. However, to date no disease-causing mutation(s) in any gene(s) have been identified. Most of the myopia loci were identified using family-based linkage analysis with microsatellite markers and the only available recent genome-wide association studies (GWAS) using SNP arrays, in a large cohort of refractive error patients identified two different loci [40,41].

The present study provides further evidence for genetic heterogeneity and indicates that more than one locus may contribute to high myopia. It also suggests that the high-grade myopia in Polish families is not allelic to any of the previously described candidate loci (personal communication, M.G.). In addition, exclusion of newly identified candidate loci in other Polish families indicates possible genetic heterogeneity within Polish population signifying that genome-wide linkage analysis in these families may reveal novel locus/loci for high myopia. Naiglin et al. [21] also reported genetic heterogeneity in families with high myopia. Earlier linkage for high myopia was reported on chromosome 7p and 12p; however these loci do not overlap with the genomic regions identified in the present family HM-32 [51,54,55]. Since we are drawing the linkage inferences from one large family with a high-density SNP data, we took proper care for accounting the false positive due to high LD, and our results were very consistent. At the same time, we have chosen the SNPs in our initial linkage panel in a way (MAF>1%) that increased the marker heterozygosity, hence, increased the linkage information content that improves the likelihood of detecting a recombinant event.

The 12.2 Mb candidate interval on chromosome 7p22.1–7p21.1 contains 61 known transcripts. These include genes involved in regulation of cell proliferation, growth and extension: β-actin (*ACTB* [OMIM 102630]), fascin homolog 1, actin-bundling protein (*FSCN1* [OMIM 602689]), ras-related C3 botulinum toxin substrate 1 (*RAC1* [OMIM 602048]), as well as in gene expression: zinc finger protein 12 (*ZNF12* [OMIM 194536]). Further, genes considered in this locus are: islet cell autoantigen 1 (*ICA1* [OMIM 147625]) and collagen, type XXVIII, alpha 1 (*COL28A1* [OMIM 609996]).

The second interesting locus identified at chromosome 7p12.3–7p11.2 that maps to a 10.9 Mb region comprises 30 known transcripts, including growth factor receptor-bound protein 10 (*GRB10* [OMIM 601523]) and epidermal growth factor receptor (*EGFR* [OMIM 131550]). *GRB10* encodes a growth factor receptor-binding protein that interacts with insulin receptors and insulin-like growth-factor receptors [55]. Based on an animal model, it has been established, that GRB10 acts as an inhibitor of intracellular signaling pathways regulating growth and metabolism. Gene knockouts in mice results in disproportionate overgrowth of the embryo and placenta [56]. EGFR and its ligands are cell signaling molecules involved in diverse cellular functions, including cell proliferation, differentiation, motility, and survival, and in tissue development [57]. EGFR is the prototypical tyrosine kinase receptor localized to basal and differentiated epithelia in the cornea and is a key regulator for maintaining a healthy cornea and promoting regrowth of the wounded cornea [58,59]. Furthermore, Domínguez et al. [60] reported various EGFR functions in *Drosophila* eye development.

The locus on chromosome 12p12.3–12p12.1 maps within 9.5 Mb region that has 32 known transcripts which are involved in cell signaling and proliferation: Rho GDP dissociation inhibitor (GDI) beta (*ARHGDIB* [OMIM 602843]), RAS-like, estrogen-regulated, growth inhibitor (*RERG* [OMIM 612664]), and epidermal growth factor receptor pathway substrate 8 (*EPS8* [OMIM 600206]), phosphoinositide-3-kinase, class 2, gamma polypeptide (*PIK3C2G* [OMIM 609001]).

In the present linkage analysis, all HM-32 family members who carried the three disease-related haplotypes were found with high-grade myopia, indicating that more than one locus contributes to the high myopia phenotype in this pedigree. It is also possible that one of these linked loci is a major dominant determinant and that the others are modifier genomic variants. Therefore, we hypothesize that the high-grade myopia phenotype in this family could be due to multifactorial inheritance; however, it is difficult to prove this hypothesis until we identify the pathologic mutations. Further research is needed to understand role of multifactorial inheritance and how high-grade myopia can be prevented and/or treated.

## Appendix

Appendix 1. Pedigrees of remaining 27 Polish families with high grade myopia. Filled symbols: individuals with high myopia; open symbols: unaffected individuals; symbols with question mark: individuals with unknown disease status. To access the data, click or select the words “Appendix 1.” This will initiate the download of a compressed (pdf) archive that contains the file.

Appendix 2. Detailed clinical findings in family HM-32. To access the data, click or select the words “Appendix 2.” This will initiate the download of a Microsoft Word (.doc) file that contains the Table.

Appendix 3. Statistically significant differences in spherical refractive error (SPH), spherical equivalent refractive error (SE), and axial length (AL) between studied groups. To access the data, click or select the words “Appendix 3.” This will initiate the download of a Microsoft Word (.doc) file that contains the Table.

Appendix 4. Detailed linkage analysis results of three distinct loci identified on chromosome 7p22.1–7p21.3, 7p12.3- 7p11.2, and 12p13.1–12p12.2, respectively (**A**-**C**) for Family HM-32. To access the data, click or select the words “Appendix 4.” This will initiate the download of a Microsoft Word (.doc) file that contains the Table.

Appendix 5. Linkage analysis for chromosome 7p22.1–7p21.1, 7p13–7p11.2, and 12p13.1–12p12.1 loci. For each locus 1 SPN per 0.5 cM, 1.0 cM, 1.5 cM, and 2 cM was selected to prune out the LD. PL – parametric linkage, NPL – non-parametric linkage. To access the data, click or select the words “Appendix 5.” This will initiate the download of a Microsoft Word (.doc) file that contains the Table.
